# Puerarin inhibits EMT induced by oxaliplatin *via* targeting carbonic anhydrase XII

**DOI:** 10.3389/fphar.2022.969422

**Published:** 2022-08-25

**Authors:** Xindong Chen, Zhiruo Zhou, Zhi Zhang, Chenhao Zhao, Jiayu Li, Jingwen Jiang, Biao Huang, Yuan Qin

**Affiliations:** ^1^ College of Life Sciences and Medicine, Zhejiang Sci-Tech University, Hangzhou, China; ^2^ School of Environmental Science and Engineering, Zhejiang Gongshang University, Hangzhou, China; ^3^ Zhejiang Provincial Key Laboratory of Solid Waste Treatment and Recycling, Hangzhou, China

**Keywords:** puerarin, oxaliplatin, breast cancer, epithelial-mesenchymal transition, carbonic anhydrase XII

## Abstract

Puerarin is a flavonoid molecule that widely exists in various plants. Puerarin has been reported to exhibit anti-tumor effects in various cancers. However, its exact underlying pharmacological mechanism is unclear. This study evaluated the anticancer effect of puerarin combined with oxaliplatin (OXA) *in vitro* and *in vivo*. Our results indicated that puerarin can reverse platinum-based anti-cancer drug resistance, and enhance the OXA’s anticancer effects on breast cancer. Furthermore, puerarin can inhibit migration and reverse the epithelial-mesenchymal transition (EMT) induced by low-dose OXA. Further studies showed that the carbonic anhydrase (CA) XII is a potential target of puerarin. In conclusion, puerarin is expected to become an adjuvant chemotherapy drug and potentially become one of the medicated foods for breast cancer patients.

## Introduction

Chemotherapy is used to treat cancer, prolong the life of cancer patients, and even cure cancer, however, sometimes it can stimulate cancer cells and cause metastasis (Wills et al., 2021; Dai et al., 2022). Therefore, continuous efforts have been made to find and develop new strategies for cancer therapy with lower side effects and better efficacy. For this, various researchers pay great attention to compounds from natural plants and their derivatives, which can be potential in the treatment of cancers (Chen et al., 2009; Shirode et al., 2015). These compounds are found in many diets and can be good options for adjuvant cancer treatment.

Flavonoids are a type of natural small molecules with anti-cancer, anti-inflammatory and antioxidant effects (Maleki et al., 2019; Bisol et al., 2020; Kopustinskiene et al., 2020; Liu et al., 2022). Puerarin is a typical flavonoid molecule and active ingredient extracted from leguminous plants of the genus *Pueraria*, an important medicinal plant known for its health and beauty benefits. In 1993, puerarin was approved for clinical use and was widely used in treating cardiovascular diseases (Ahmad et al., 2020). In addition, puerarin has two benzene rings (A ring and B ring) linked to each other through the central three carbon structure, and many evidences have proved that this structure has the ability to down-regulate mutant p53 protein, block cell cycle and inhibit Ras protein expression and anti-cancer properties (Lamson and Brignall, 2000; Bisol et al., 2020). Various studies demonstrated that puerarin plays an anti-cancer role in several trials (Wang et al., 2013; Kang et al., 2017; Liu et al., 2017). Puerarin inhibits lymphatic carcinoma cell proliferation and reduces the levels of matrix metalloproteinase through reactive oxygen oxidative stress. In addition, through endogenous and exogenous mitochondrial pathways, puerarin induces tumor cells apoptosis (Chen et al., 2016; Hu et al., 2018). Puerarin selectively reduces tumor cells’ proliferative capacity and extensively inhibits cancer cells signaling pathway transduction (Liu et al., 2017). Liu et al. showed that lipopolysaccharide (LPS) treatment increases the capacity to metastasize of breast cancer cells, while puerarin reduces the metastasis and invasion of LPS-induced breast cancer cells (Liu et al., 2017), suggesting that puerarin can potentially be used for anti-breast cancer.

Epithelial-mesenchymal transition (EMT) refers to how epithelial cells are depolarized and transformed into mesenchymal cells due to certain factors (Jiang et al., 2022). During the metastasis in epithelial tumors, the phenotype of tumor cells changes primarily caused by environmental stimuli that enable tumor cells to adapt to the various microenvironments they encounter (intercellular stroma, humoral components, or blood) (Park et al., 2020; Qiao et al., 2021). EMT regulates these phenotypic transformations. Therefore, to some extent, EMT promotes tumor metastasis (Pastushenko and Blanpain, 2019). Meanwhile, it has been reported that low-concentration chemotherapy drugs not only significantly inhibit tumor proliferation, but also induce EMT of tumor cells, thus promoting tumor metastasis (Middleton et al., 2018).

Therefore, this study aims to detect whether puerarin can enhance the effect of oxaliplatin (OXA), the third generation of platinum chemotherapy drugs, on breast cancer and inhibit metastasis of breast cancer cells.

## Materials and methods

### Chemicals and cell culture

Puerarin was purchased from Meilunbio (Dalian, China). E-cadherin antibody (ab40772) and vimentin antibody (BF8006) were purchased from Abcam and Affinity, respectively. Apoptosis Detection Kit was purchased from Beyotime (Shanghai, China). Crystal violet was purchased from Sigma-Aldrich Fluka (America). OXA was purchased from Meilunbio (Dalian, China), LTD. Cisplatin (DDP) was purchased from Sigma-Aldrich.

The MCF-7 (human breast cancer cell lines) and MCF-7/DDP (DDP-resistant cell lines), were from KeyGEN BioTECH (Nanjing, China). These cells were grown in DMEM containing penicillin, streptavidin, and 10% bovine serum at 5% CO_2_, and 37°C.

### Cell viability assay

The drug tolerance effects of puerarin were detected using the 3-(4,5-dimethyl-2-thiazolyl)-2,5-diphenyl-2-H-tetrazolium bromide (MTT) assay on MCF-7, MCF-7/DDP. The cells (5,000 cells/well) were added to 96-well plates cultured overnight. All assays were repeated three times. In each flask, MTT solution was added after 48 h of drug treatment. Incubation in dimethyl sulfoxide (DMSO) for 4 h dissolves the formazan crystals. OD_590_ value was detected with a microplate reader, and detected a 50% inhibitory concentration (IC_50_) value. The synergistic effect was calculated using CompuSyn software.

### Rh123 efflux assay

Cells (1 × 10^6^) were cultured for 24 h in six-well plates. A variety of levels of puerarin were used for the pretreatment of MCF-7/DDP cells for 24 h [0 μM, 20 μM (L), 40 μM (H)]. After the pretreatment, cells were incubated with Rh123 (5 mg/mL) in a dark room. Then, Rh123-free medium was used to replace the above medium, and drained the remaining efflux intervals every hour. After incubation, PBS was used to wash cells twice. Then, 400 μL lysis buffer was used for lysing cells, and PBS with 10% FBS was used to maintain the cells. The flow cytometry was used to determine the green fluorescence of Rh123.

### Wound-healing assay

A wound healing assay was performed to assess changes in cell motility and migration. Cells were grown to confluency at 5 × 10^5^ cells per well in 48-well plates. Scratch the cell monolayer with an apipette tip and then rinse using phosphate buffer saline (PBS). After the treatment [Control, OXA (5 μM), puerarin (40 μM) + OXA (5 μM)], a Nikon microscope was used to take images 0, 24, and 48 h.

### Transwell assays

The transwell assay is used to evaluate cell invasiveness. Three different concentrations of medium [Control, OXA (5 μM), puerarin (40 μM) + OXA (5 μM)] were used for the suspension of the cells below. The cells were then inoculated into an 8 μm polyethylene terephthalate filter membrane coated with matrix gel. In the lower chamber, about 500 mL of medium was placed. The cells were fixed for 30 min with paraformaldehyde (4%) and stained for 20 min with crystal violet (0.1%). A hundred-fold magnification inverted microscope was used to image the invaded cells, and the cell numbers were manually counted.

### Immunofluorescence assays

Cells were treated [Control, OXA (5 μM), puerarin (40 μM) + OXA (5 μM)] and were cultured overnight. The cells were treated with methanol and Triton X-100. E-cadherin and vimentin (both 1:100) were used in an immunofluorescence experiment. After washing thrice for 20 min, Incubation of the cells with a secondary antibody (1:200) for 30 min. Following 4,6-diamino-2-phenyl indole (DAPI) staining for 10 min, and observed under a laser scanning confocal microscope.

### Apoptosis assays

A 96-well plate was used to culture the cells. After drugs treatment, the culture solution was discarded. Then, Annexin V-FITC binding solution, Annexin V-FITC and propyl iodide solution were successively added and gently mixed all the solutions. In a dark room, plates were incubated for 20 min. The red and green fluorescence was detected by a fluorescence microscope.

### Animal studies

In this section, BALB/c nude mice (5 weeks) were used. All mice were raised in sterile conditions. All procedures were approved according to the guidelines of the Animal Ethics Committee of the Zhejiang Sci-tech University. The MCF-7 cells were orthotopically implanted into the mice. Cells were grown until the logarithmic growth stage, centrifuged with PBS. 50% Matrigel mixture was resuspended in PBS, resulting in 2 × 10^7^ cells/mL. The right flank of each mouse was injected 0.2 mL cell suspension. After 14 d of tumor transplantation, four groups of mice were divided: control group (saline given orally once daily), puerarin treated group (50 mg/kg), OXA treated group (5 mg/kg), and puerarin and OXA treated group (50 mg/kg + 5 mg/kg). After the tumor was transplanted, daily measurements were taken of its volume and weight. All mice were euthanized after 3 weeks. Tumors were resected and volumes were measured. V = ab^2^/2 (a = length, b = width) was used to calculate tumor volumes. To measure their survival rates, another 40 mice were distributed into 4 groups (10 per group). The survival time of each mouse was monitored.

### RNA sequencing data collection and procession

The RNA sequencing data for cancer tissues and adjacent tissues from patients with breast cancer were obtained from the Gene Expression Omnibus (GEO) database. The differentially expressed gene (DEG) was considered by four sample data, involving GSM2286198, GSM2286199, GSM2286316, and GSM2286317 with two drug treatment samples and two control samples.

### Analysis of differentially expressed genes

In the R computing environment, with the Limma package, the corresponding fold change and *p* value for DEGs between different groups were compared using a volcano plot. Up- and down-regulated genes had *p* ≤ 0.05, a fold change of more than 2.0, defined as log_2_ (fold change) > 1 or < - 1 for up- and down-regulated genes, respectively. For gene function enrichment analysis, gene ontology (GO) annotations of genes in R software were used. A maximum gene of 5,000 and a minimum gene of 5 was set (*p* < 0.05 and FDR <0.25).

### Target prediction and molecular docking

To predict puerarin’s targets, the simplified molecular input line entry system (SMILES) format of puerarin from PubChem and the similarity ensemble approach (SEA) website were used (Keiser et al., 2007). Molecular docking was performed with puerarin and carbonic anhydrase (CA) XII. An analysis of the complex between the ligand and protein was carried out by Pymol using the PDB format.

### CA XII activity analysis

The CA XII activity in breast cancer cells was determined by extracellular pH analysis. Cells were treated with [20 μM (L) puerarin +500 nM U-104] and [40 μM (H) puerarin +200 nM U-104] for 3 h. The Wilbur-Anderson method was used to calculate CA XII activity (WAU/mg = 2× (T_0_-T)/T*mg protein). To determine how long it will take to reduce the pH of an isotonic buffer from 8.00 to 6.60, using time (T) (T: catalyzed reaction and T0: unanalyzed reaction).

### Data statistics

Data are analyzed as mean ± standard deviation (SD). The independent variance *t*-test were used to assess the differences between the two groups. The multiple comparisons test were compared using a one-way analysis of variance, followed by the least significance difference (LSD) post-hoc test (SPSS 23.0).

## Results

### Puerarin enhanced the effects of platinum-based anti-cancer drug and reversed drug-resistance

**FIGURE 1:**
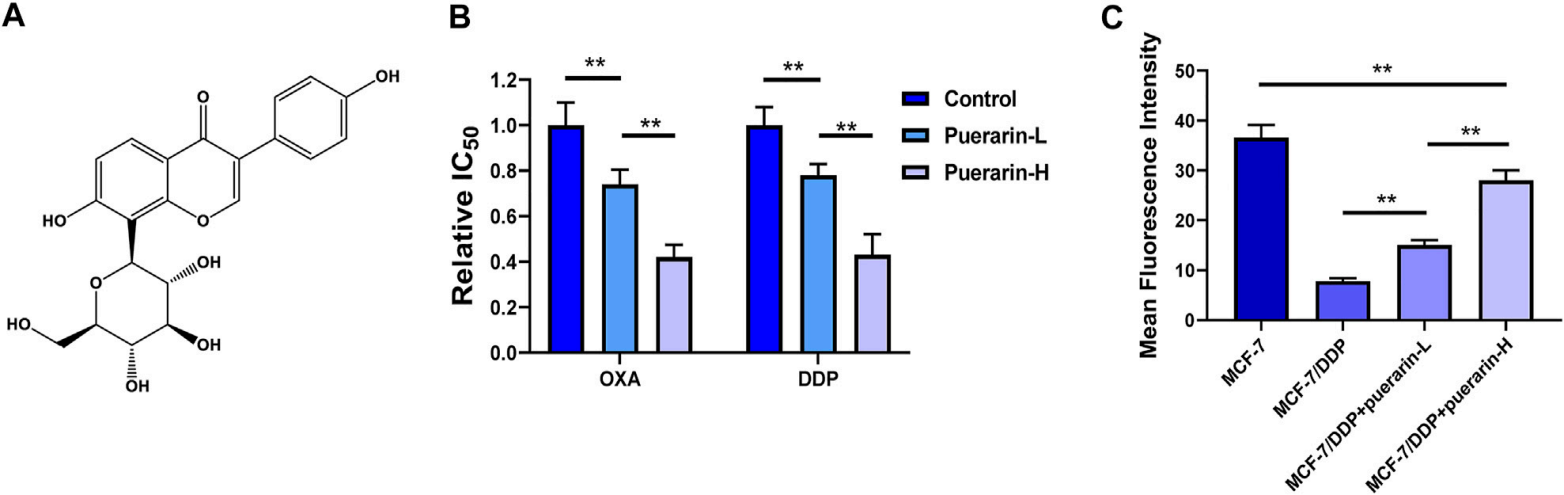
Puerarin reversed drug resistance of platinum-based anti-cancer drug. **(A)** The structural formulas of puerarin **(B)** The IC_50_ values of OXA and DDP on MCF-7 were detected. The cancer cells were treated with different concentrations of puerarin (Control: DMSO, puerarin-L: 20 μM, puerarin-H: 40 μM). **(C)** The fluorescence intensity of Rh123 in MCF-7/DDP was lower than the fluorescence intensity in MCF-7. Puerarin treatment reversed the change in a dose-dependent manner (*p < 0.05, **p < 0.01).

Figure 1A shows the structural formulas of puerarin. In order to detect the effect of puerarin in reversing drug resistance, OD_590_ was measured. In our results, drug resistance to DDP and OXA can be reversed by puerarin in MCF-7/DDP cells. In addition, there is a dose-effect relationship in the reverse effect of puerarin on MCF-7/DDP cells (Figure 1B). A study was carried out to evaluate the efflux of Rh123 from MCF-7/DDP cells. Figure 1C indicates that as compared to the other groups, the puerarin groups had a higher fluorescence intensity.

### There is a synergistic effect of puerarin and OXA on tumor inhibition

**FIGURE 2:**
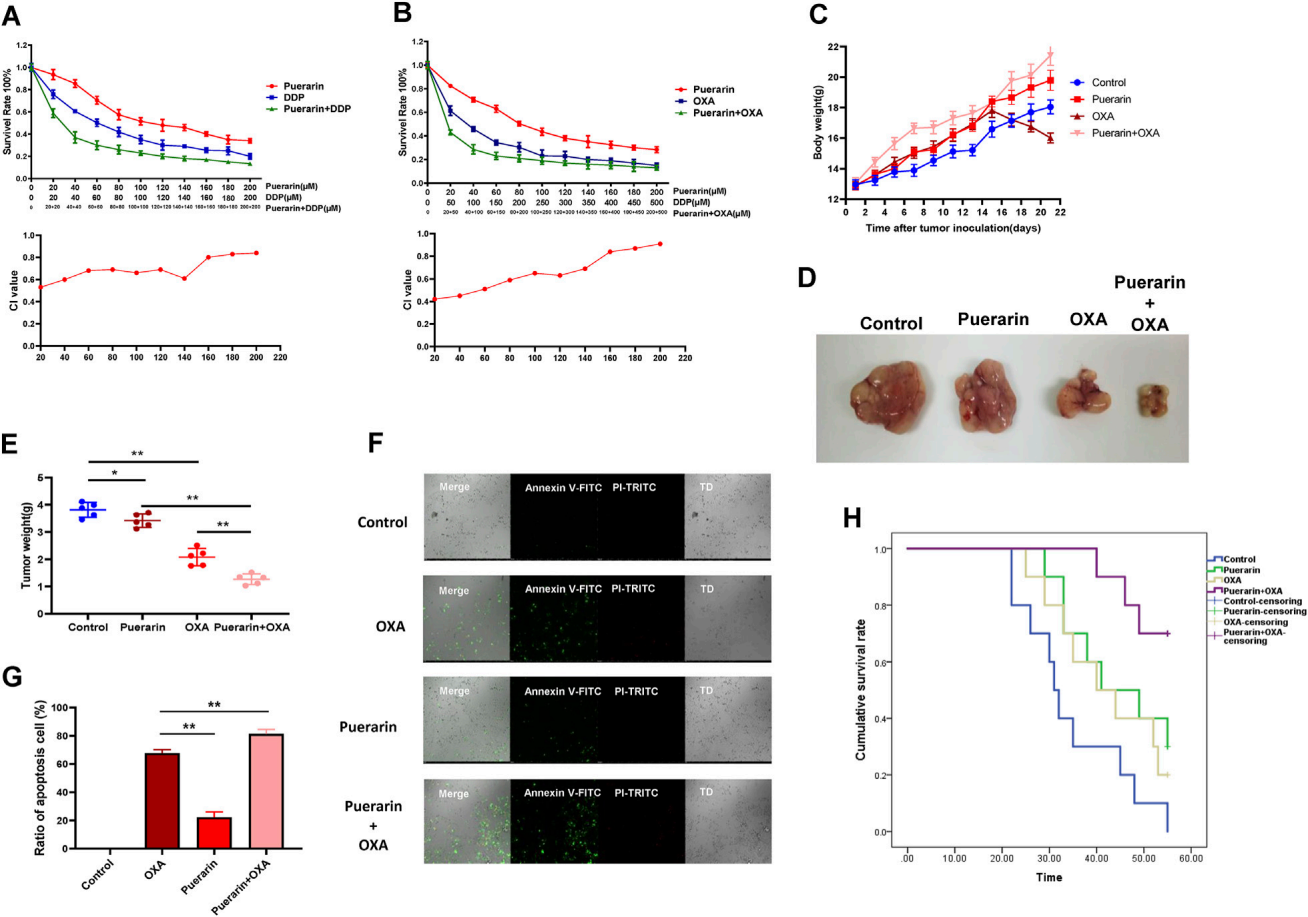
Puerarin enhanced the effects of OXA. **(A,B)** MTT assay was operated to detect the inhibition on MCF-7 cells. Puerarin enhances the inhibitory effect of OXA and DDP on cell proliferation **(C–E)** Mice in the combination treatment group gained weight compared to those in the OXA-treated group. Compared to the other groups, the tumor weight of co-treatment group is lowest. **(F,G)** Annexin V-FITC and PI-TRITC were used to detected the apoptosis cells. Puerarin improves the efficacy of OXA and a significant increase in apoptosis cells was observed in the co-treatment group **(H)** Mice of co-treatment group had a higher survival rate than the mice in other groups (*p < 0.05, **p < 0.01).

In order to detect the synergistic effects of puerarin and OXA, the MTT assay was performed. As shown in Figures 2A,B, puerarin improves the inhibitory effect of OXA and DDP on cell proliferation. Furthermore, combining puerarin with platinum-based drugs resulted in a combination index (CI) value less than 1, thus indicating synergistic effects. The effect of co-treatment on MCF-7/DDP xenografts in nude mice BALB/c was evaluated. Mice in the combination treatment group gained weight compared to those in the OXA-treated group. Compared to the other groups, the tumor weight of co-treatment group is lowest (Figures 2C–E).

Each group of tumor cells was tested for apoptosis using the apoptosis detection kit. Annexin V was marked with green fluorescence indicating apoptotic cells and PI was marked with red fluorescence indicating necrotic cells. As shown in Figures 2F,G, puerarin improves the efficacy of OXA and a significant increase in apoptosis cells was observed in the co-treatment group. Moreover, compared to a single treatment, mice of co-treatment group had a higher survival rate (Figure 2H).

### Puerarin inhibited migration and invasion and reversed EMT induced by low dose OXA

**FIGURE 3:**
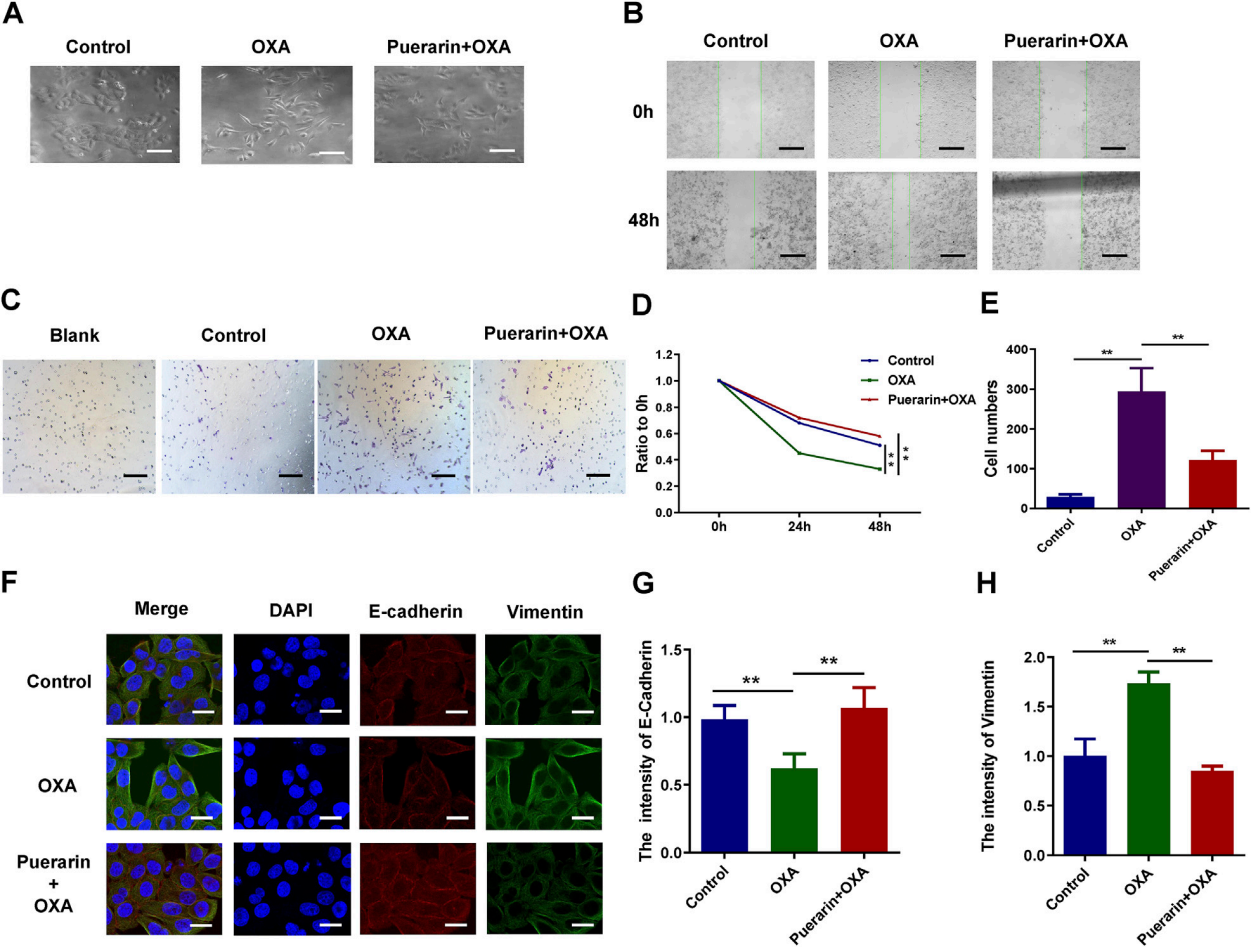
Puerarin inhibited migration and invasion and reversed EMT. **(A)** Morphological changes of the cancer cells in different treatment groups observed by a microscope **(B,D)** The results of wound-healing assay. **(C,E)** The results of transwell assay **(F–H)** The results of immunofluorescence (*p < 0.05, **p < 0.01).

Morphological changes in the cancer cells in different treatment groups were observed by an optical microscope. As a result of low dose OXA, cancer cells developed pseudopodia and the co-treatment group displayed signs of apoptosis, such as rounded and shed cells, as shown in Figure 3A. The migration and invasion of co-treated cells was detected by wound-healing assay and transwell assay, and the results as shown in Figures 3B,D, the migration was highly enhanced in the OXA-treated group, whereas, it was significantly inhibited in the co-treatment group. Similarly, A combination of purarin and OXA inhibits the invasion of cancer cells by low-dose OXA (Figures 3C,E). Moreover, the expression of EMT biomarkers was detected using an immunofluorescence assay and it was found that vimentin levels of co-treatment group were lower than that of OXA group, whereas E-cadherin levels were higher, as shown in Figures 3F–H. Based on these results, the OXA-treated group promotes EMT, whereas puerarin inhibits the EMT process caused by OXA.

### There is a widespread influence of puerarin in cancer cells

**FIGURE 4:**
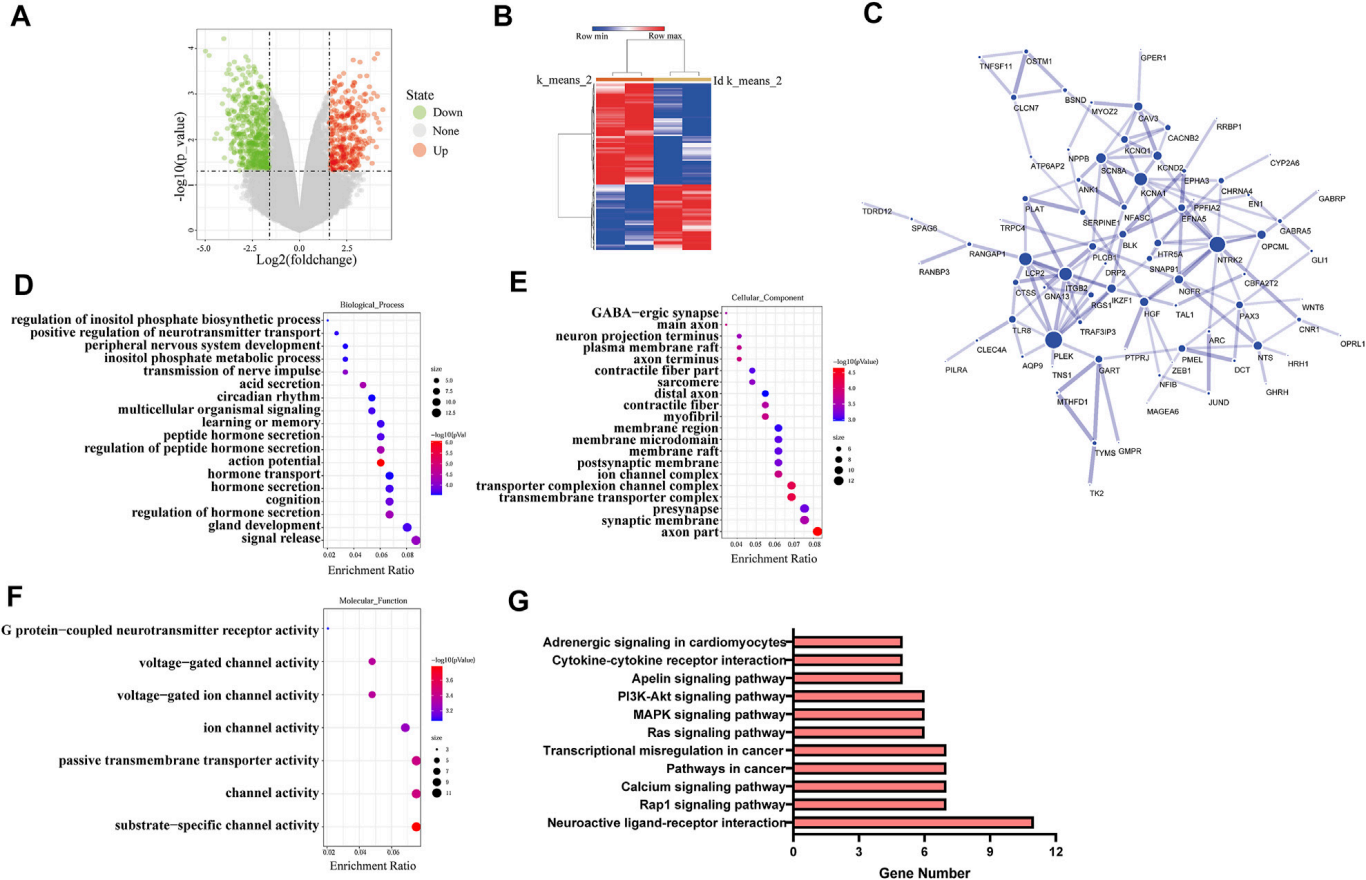
Puerarin effected multiple functions and signal pathways of cancer cells. **(A)** The volcano plot of differentially expressed genes **(B)** The hierarchical clustering of differentially expressed genes. **(C)** The protein-protein interaction network of differentially expressed genes **(D–F)** The GO analysis results of differentially expressed genes, **(D)** Biological process, **(E)** Cellular component, **(F)** Molecular fuction **(G)** The KEGG analysis results of differentially expressed genes.

The RNA sequencing data (GSE85871) for cancer tissues and adjacent tissues of patients with breast cancer were got from GEO database. The graph was drawn with the log_2_ ratio and the −log_10_ (*p*) of each gene as shown in Figure 4A. Downregulated genes and upregulated genes are indicated by green and red, respectively. The hierarchical clustering was made using the differentially expressed genes, as shown in Figure 4B. The various interactions between the control group and puerarin treatment groups were recorded by the STRING database and visualized in Cytoscape, as shown in Figure 4C. Analysis with GO and the KEGG revealed that differentially expressed genes tended to be enriched for tumor metastasis and energy metabolism, as shown in Figures 4D–G.

### CA XII is a potential drug target of puerarin

**FIGURE 5:**
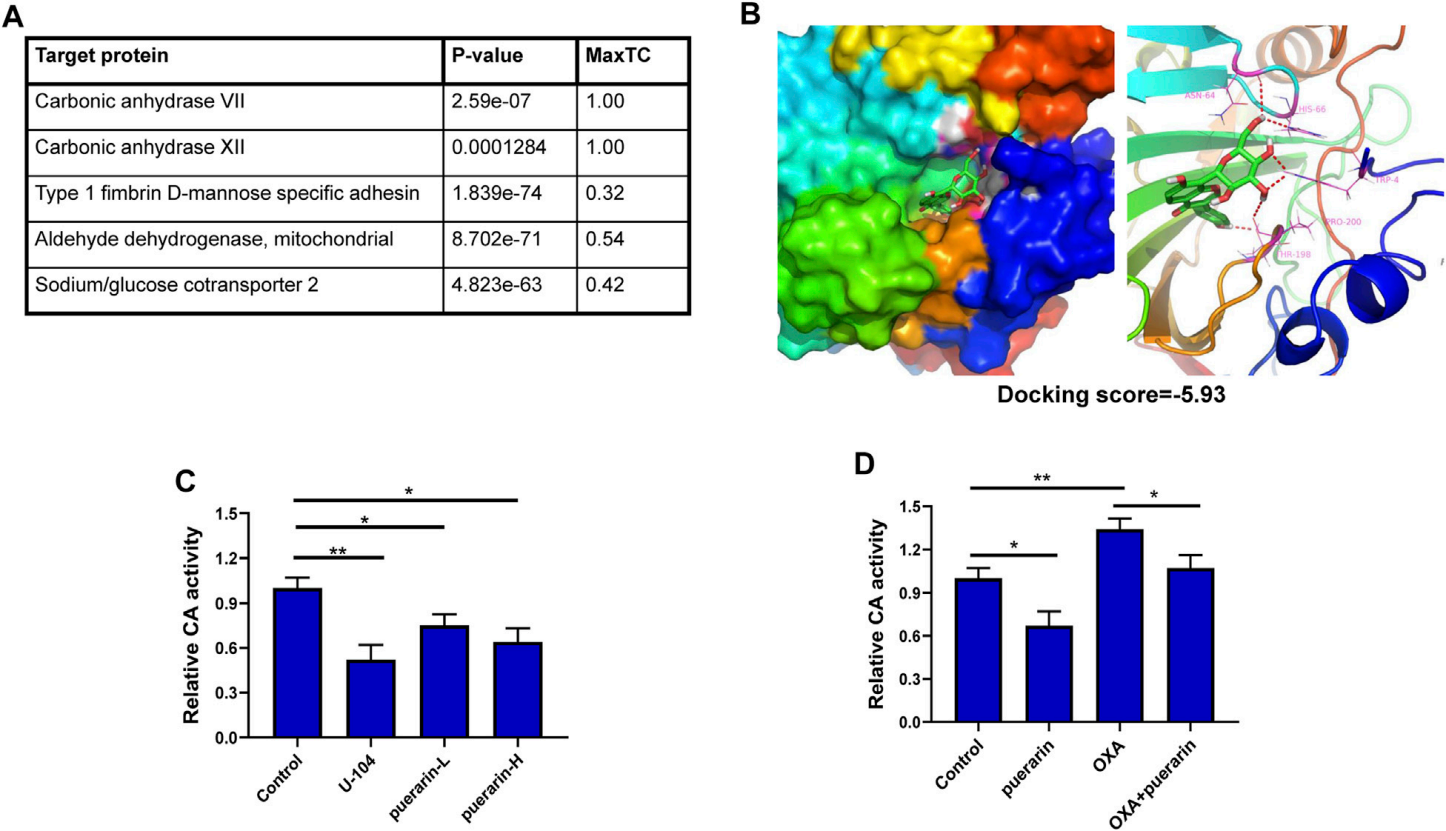
CA XII is a potential drug target of puerarin. **(A)** The predicted targets for puerarin **(B)** The molecular docking results showed that there is a good combination between puerarin and CA XII, with the docking score -5.93. **(C)** Puerarin could inhibit CA activity under a dose-effect relationship **(D)** Low dose OXA treatment increased the extracellular pH level compared with the control group, and puerarin inhibited this trend (*p < 0.05, **p < 0.01).

The SMILES format of puerarin from PubChem and the SEA website was used to predict puerarin’s targets. CA VII and CA XII were the first two potential drug targets with the Max Tanimoto Coefficient (MaxTC) of 1.00, as shown in Figure 5A. The CA XII is more closely associated with cancer cells than with CA VII. The molecular docking results showed that there is a good combination between puerarin and CA XII, with the docking score -5.93, as shown in Figure 5B. CA XII catalyzes the hydration of carbon dioxide to H^+^ and HCO_3_^−^, causing the acidic extracellular pH to decrease. The ability of puerarin to inhibit CA activity was determined by measuring extracellular pH. The results showed that puerarin could inhibit CA activity under a dose-effect relationship (Figure 5C). Low dose OXA treatment increased the extracellular pH compared to the control group, meanwhile, the puerarin inhibited this trend, thereby suggesting that puerarin inhibited CA activity, as shown in Figure 5D.

## Discussion

Recently, for breast cancer, while significant progress has been made in diagnosis and treatment, the prognosis remains bleak (Zhao et al., 2017; Chen et al., 2022). It was reported that after OXA treatment, residual cancer cells revealed increased metastasis significantly. Our previous study and other research all showed that low-dose platinum-based anti-cancer drugs could induce EMT of cancer cells (Liu et al., 2015). EMT caused by chemotherapy is a significant determinant of drug resistance to chemotherapy and cancer metastasis.

New therapeutic options that safely enhance chemotherapy sensitivity significantly improve efficiency in cancer treatment (Luan et al., 2020; Zhang et al., 2020). In our study, the combination of puerarin and OXA can improve the sensitivity of OXA chemotherapy, thus, inhibiting the metastasis of breast cancer. In addition, puerarin can reverse OXA resistance in drug-resistant breast cancer. The combined administration of puerarin can also inhibit low-dose OXA-induced EMT as indicated in the results. Meanwhile, the co-treatment group inhibited tumor weight *in vivo* compared with the chemotherapy drug group alone. Therefore, puerarin can be used as a complementary medicine for OXA in enhancing the chemotherapy sensitivity and anti-cancer ability of OXA.

Multiple studies have shown that puerarin has good anticancer mechanisms against several cancer cells (Li et al., 2019; Aboushanab et al., 2021). However, the exact molecular mechanism and potential drug target of puerarin remain unknown (Wang et al., 2020). Natural small molecules from plants generally have an extensive range of pharmacological activities (Sun et al., 2017). Our results showed that puerarin has the potential to inhibit various functions and signaling pathways of breast cancer cells. Furthermore, it was found that the CA XII is the puerarin’s potential target and puerarin inhibits the acid secretion mediated by the CA protein. The CA XII exists in various organsand plays a key role in life activities (Karhumaa et al., 2000; Parkkila et al., 2000; Liao et al., 2003). CA XII’s expression can be detected in various types of tumor, including breast cancer and other cancer (Kivela et al., 2005; Hsieh et al., 2010; Ilie et al., 2011). Extensive evidences suggest that CA XII plays a key role in the migration, invasion and metastasis of cancer cells. Hsieh et al. demonstrated that silencing CA XII also reduces the migration and invasion of breast cancer cells, and that CA XII interacts with matrix metalloproteinases in proteolysis of ECM during migration and invasion of cancer cells (Hsieh et al., 2010). As another target protein in this study, CA VII is mainly related to the pathogenesis of neuromuscular disorders and has almost no correlation with cancer (Pastorekova et al., 2004; Viikilä et al., 2016), therefore we chose CA XII as our target protein. Recent studies have indicated that CA XII participates in chemotherapy drug resistance, hence, promoting the further development of tumors (Kobayashi et al., 2012; Kopecka et al., 2015). Our results showed that a low dose of OXA activates the CA XII’s activity in breast cancer cells, which is detrimental to cancer treatment. Puerarin can inhibit the activity of CA XII, which can influence chemotherapy drugs to enhance the anticancer effect of chemotherapy drugs.

There are also some deficiencies in this study. In future research, in-depth studies on the mechanism of puerarin *in vivo* and systematic studies on the toxicity, side effects of puerarin could be done. In addition, the pharmacological activity of puerarin could be further enhanced by targeted modification of its structure through medicinal chemistry methods.

Our results demonstrated the anticancer effects of puerarin on tumor cells and models of xenograft mice. Puerarin targeted CA XII and affected multiple carcinogenic signaling networks. In addition, puerarin significantly increased platinum sensitivity and inhibited platinum-induced EMT in breast cancer. There are some reasons why puerarin is expected to become an adjuvant chemotherapy drug and has the potential to become one of the medicated foods for breast cancer patients.

## Data Availability

The datasets presented in this study can be found in online repositories. The names of the repository/repositories and accession number(s) can be found in the article/Supplementary Material.
